# Neural correlates of RDoC-specific cognitive processes in a high-functional autistic patient: a statistically validated case report

**DOI:** 10.1007/s00702-021-02352-w

**Published:** 2021-05-18

**Authors:** Laura S. Daedelow, Anne Beck, Lydia Romund, Lea Mascarell-Maricic, Isabel Dziobek, Nina Romanczuk-Seiferth, Torsten Wüstenberg, Andreas Heinz

**Affiliations:** 1 Department of Psychiatry and Psychotherapy CCM, Charité–Universitätsmedizin Berlin, corporate member of Freie Universität Berlin, Humboldt-Universität Zu Berlin, and Berlin Institute of Health, Berlin, Germany; 2 Health and Medical University Potsdam, Potsdam, Germany; 3 Berlin School of Mind and Brain, Berlin, Germany; 4 Department of Psychology, Humboldt-University of Berlin, Berlin, Germany; 5 Department of Clinical Psychology and Psychotherapy, Psychological Institute, Ruprecht-Karls-University Heidelberg, Hauptstr. 47–51, 69117 Heidelberg, Germany

**Keywords:** Autism spectrum disorder, Research Domain Criteria, Emotion processing, Reward processing, Working memory, Neuroimaging

## Abstract

The level of functioning of individuals with autism spectrum disorder (ASD) varies widely. To better understand the neurobiological mechanism associated with high-functioning ASD, we studied the rare case of a female patient with an exceptional professional career in the highly competitive academic field of Mathematics. According to the Research Domain Criteria (RDoC) approach, which proposes to describe the basic dimensions of functioning by integrating different levels of information, we conducted four fMRI experiments targeting the (1) social processes domain (Theory of mind (ToM) and face matching), (2) positive valence domain (reward processing), and (3) cognitive domain (N-back). Patient’s data were compared to data of 14 healthy controls (HC). Additionally, we assessed the subjective experience of our case during the experiments. The patient showed increased response times during face matching and achieved a higher total gain in the Reward task, whereas her performance in N-back and ToM was similar to HC. Her brain function differed mainly in the positive valence and cognitive domains. During reward processing, she showed reduced activity in a left-hemispheric frontal network and cortical midline structures but increased connectivity within this network. During the working memory task patients’ brain activity and connectivity in left-hemispheric temporo-frontal regions were elevated. In the ToM task, activity in posterior cingulate cortex and temporo-parietal junction was reduced. We suggest that the high level of functioning in our patient is rather related to the effects in brain connectivity than to local cortical information processing and that subjective report provides a fruitful framework for interpretation.

**Supplementary Information:**

The online version contains supplementary material available at 10.1007/s00702-021-02352-w.

## Introduction

Autism spectrum disorder (ASD) is a pervasive developmental disorder characterized in the DSM-5 as a condition with impairments in social interactions and communication as well as the presence of restricted interests and behaviours (American Psychiatric Association 2013). ASD profoundly affects professional life with high unemployment rates of more than 40% in individuals with ASD and normal intelligence (Howlin 2013; Vogeley et al. 2013; Kirchner and Dziobek 2014) and up to 95% in autistic individuals with intellectual disabilities (Howlin et al. 2004).

Here, we present the case of the female patient P.K., who despite being diagnosed with ASD, exhibits an extraordinary professional career in academic research in the highly competitive research area of Mathematics.

Although reports about remarkable cognitive and perceptual strengths in ASD are not uncommon (Mottron et al. 2006; Wheelwright et al. 2006; Baron-Cohen et al. 2009; Vital et al. 2009; e.g., Assouline et al. 2012; Gonzalez et al. 2013), the ability of patients to use these strengths for a successful career is rare (e.g., Hendricks and Wehman 2009; Kirchner and Dziobek 2014). Given this patient’s extraordinary academic success, in addition to investigating mental domains commonly impaired in ASD, we examined potentially supporting cognitive and affective factors with regard to compensatory effects (Livingston and Happé 2017).

The Research Domain Criteria (RDoC) project of the National Institute of Mental Health offers a standardized approach for the assessment of key affective and cognitive behavioural dimensions and their neurobiological correlates and suggests that alterations in these dimensions can help to identify common neurobiological mechanisms in mental disorders (Insel et al. 2010). Currently, six RDoC domains have been described in detail: (1) Positive valence systems, (2) Negative valence systems, (3) Cognitive systems, (4) Systems for social processes, (5) Arousal/regulatory systems, and (6) Sensorimotor systems (National Advisory Mental Health Council Workgroup on Changes to the Research Domain Criteria Matrix 2018). While ASD-related impairments within these domains have been intensely investigated on a behavioural level, our knowledge about underlying neural processes is limited to date (e.g., Yamada et al. 2012; Rosenblau et al. 2016; Watanabe and Rees 2017; Baron-Cohen and Lombardo 2017).

Due to the patient's interest in our research and the willingness to cooperate with us, we got the opportunity to investigate this rare case by means of functional magnetic resonance imaging (fMRI).

We focussed our investigation on three out of the five domains: first, the Positive valence system represents responses to different motivational situations, e.g., reward responsiveness. Secondly, the Cognitive system is responsible for complex cognitive processes including working memory. Thirdly, the System for social processes mediates responses within interpersonal contexts such as implicit facial emotion processing as well as making judgments about the mental state of others to interpret their behaviour (Insel et al. 2010; Cuthbert 2014, 2015).

These three domains have been chosen because of the dominant role social interaction impairments play in ASD (Lai et al., 2017; Drimalla et al., 2020), and because both working memory and reward anticipation have been associated with cognitive capacities (Alloway and Alloway 2010; Schlagenhauf et al. 2013; Gajewski et al. 2018; Kaminski et al. 2018), which may help our patient to achieve her high professional success. As our patient did not display comorbid symptoms of an affective, anxiety or motor disorder, we did not examine the Negative valence or the Arousal/regulatory or sensorimotor systems.

In ASD, there is experimental evidence on reduced activity within the Positive valence system. In the ventral striatum—an important brain area contributing to the positive valence system—Dichter et al. (2012a; b) observed reduced brain response in the nucleus accumbens (NAc) during the expectation of monetary gain in participants with ASD. In a study by Richey et al. (2014), both patients with ASD and patients with social anxiety disorder displayed a decreased NAc response during the anticipation of a social reward. This finding suggests an interaction between the valence and social processing systems.

Regarding the Cognitive system, a series of studies in children and adults with ASD observed impairments in several cognitive domains of executive functioning, including working memory, planning, cognitive flexibility, inhibitory control, and action monitoring (for a review see Demetriou et al. 2019). Brain imaging studies observed altered functional activation among persons with ASD in brain areas implicated in these cognitive capacities including the dorsolateral prefrontal cortex, the dorsal anterior cingulate cortex and the posterior cingulate cortex (Luna et al. 2002; Di Martino et al. 2009).

Regarding the System for social processes and in line with the characteristic social impairments of ASD, a multitude of studies described impairments in socio-emotional and socio-cognitive domains, e.g., in face perception or mentalizing (Schultz et al. 2000; e.g., Grelotti et al. 2002; Kliemann et al. 2012). Imaging studies observed hypoactivation and altered cortical volume of the fusiform face area in subjects with ASD, which can contribute to impairments in the perception of emotional facial expressions (Schultz et al. 2000; Pierce 2001; Piggot et al. 2004; Wang et al. 2004; Dziobek et al. 2010). Deficits in social cognition in persons with ASD have mainly been associated with reduced activation of the medial prefrontal cortex, the temporo-parietal junction (TPJ), the temporal poles as well as the amygdala (Castelli et al. 2002; for a review see Dichter 2012).

These findings suggest that behavioural alterations in ASD are associated with distinguishable neural dimensions in accordance with the RDoC approach. Between and within subject variance along such dimensions may help to map patterns of individual impairments and strengths. Individual differences in such behavioural and neurobiological dimensions require careful matching of experimental samples regarding the symptoms of interest and can render group-level comparisons within different RDoC domains difficult or even impossible. Therefore, careful investigation of a single case with high-functional autism can help to identify specific alterations contributing to both specific symptoms as well as abilities. Our study has a dual aim: first, we are interested in comparing the neurocognitive and neuroimaging data of a single female highly functional patient with ASD to a demographically comparable control sample of healthy female subjects to identify the individual neurobiological markers of the RDoC domains System for social processes, Positive valence system and Cognitive system. Secondly, we aim to overcome the methodological limitations of usually descriptive case reports. Crawford and Garthwaite (2002) have shown that adjusted *t*-tests can be used to make statistical inferences about the relationship between the case and control populations.

Due to the exploratory nature of this study, we can only make broad assumptions about the expected results. Nevertheless, given the successful professional career of our case we expect similar or even superior behavioural performance to the control group in all domains. Due to the probably increased effort to perform well in the tasks associated with social processes (ToM, face matching), on the neural level we assume an increase in brain activity (Dichter 2012). In contrast, we expect no differences or a decrease in brain activity for the tasks associated with the domains of the Positive valence system (Reward task; Dichter 2012) and the Cognitive system (N-back task) (Luna et al. 2002; Di Martino et al. 2009).

## Methods and materials

### Participants

All female participants were enrolled in a large multi-centre study on neurobiological correlates of different cognitive and socio-emotional functions (http://www.ngfn-moods.de/.de/en/schizophrenie.html). The present analyses focussed on a subsample of subjects measured at the Charité–Universitätsmedizin Berlin. The study was performed in accordance with the latest version of the Declaration of Helsinki and approved by the local Ethics Committee of the Charité – Universitätsmedizin Berlin (Approval-No.: EA1/163/08). Subjects participated in the study after providing written informed consent as previously described (Esslinger et al. 2009).

#### Case patient

Female patient, 48-years-old, with excellent mathematical abilities and a top-ranking academic job in this field. The diagnosis of ASD was given according to DSM-5 criteria based on a clinical interview and the Autism Diagnostic Observation Schedule-2 (ADOS-2, Lord et al. 1999), which has high sensitivity, specificity and predictive value (Lord et al. 1999; Hus and Lord 2014) for Asperger syndrome and autism without mental retardation. The ADOS-2 calibrated severity scores of the patient were as follows: overall index (12), communication (5), and social interaction (7). In addition, the Adult Asperger Assessment (AAA, Baron-Cohen et al. 2005) was performed. Parental informants were deceased, thus no Autism Diagnostic Interview-Revised (ADI-R, Lord et al. 1994) was performed. The Autism Spectrum Quotient (AQ = 42) and Empathy Quotient (EQ = 7) were assessed. Clinically, diagnosis was confirmed by two specialists in adult psychiatry (Imke Puls and Andreas Heinz) and a specialist in Children and Adolescent Psychiatry (Jakob Hein). Handedness was measured using the Edinburgh Handedness Inventory (Oldfield 1971), confirming right-handedness.

#### Control group

The control group consisted of 14 healthy women aged 40–50 years (*M* = 45, *SD* = 2.43), who did not report any neurological disorder or lifetime psychiatric Axis I disorder including drug or alcohol dependence in a clinical interview and standardized assessment according to the Screening Interview for DSM-IV Axis I Disorders (SCID I, First et al. 2002). Regarding handedness, 2 control subjects were left-handed, the remaining 12 were right-handed.

### Psychometrics

All participants were investigated using neuropsychological testing as well as personality inventories: assessment of crystalline intelligence was performed using the German version of the multiple-choice verbal intelligence test (MWT-B; Lehrl 2005); for the assessment of fluid intelligence, we used the matrices test taken from the German “Hamburg-Wechsler-Intelligenztest für Erwachsene” (HAWIE-R; Tewes 1991). Moreover, we applied the NEO Five-Factor Inventory (NEO-FFI; Costa and McCrae 1992) as well as the Temperament and Character Inventory (TCI; Cloninger 1994).

### Triadic dimension

In our opinion, the knowledge about the perspective of the patient can add essential information for an adequate interpretation of experimental findings. Therefore, we offered the patient to report her own impressions concerning the general experimental set up (scanner environment, etc.) and the specific empirical paradigms, which we provide in the results section.

### RDoC domain specific experimental paradigms

#### Positive valence system—Reward task

In the Reward task, subjects were instructed to respond as fast as possible to a bright flash of light via button press. Four different cues were presented before the flash indicating whether a monetary reward would follow. The first cue was a vertically oriented arrow pointing upward, presented for 6 s and immediately followed by the flash of light. After the button press, a verbal feedback (“fast” or “slow”) together with the information about the earned amount (either 2 € or 0 €) was given. The second cue was a vertically oriented arrow pointing downward presented in the same way like to first cue, only in this condition subjects could lose money (− 2 €) or avoid losing by a fast button press (0 €). The third cue consisted of a vertically oriented double-sided arrow. The difference to the other two conditions was that the feedback contained no information about a monetary gain. Finally, a horizontally oriented double-sided arrow was presented, followed by a black screen for 3 s to include a control condition without any anticipation of a consequence. The inter-trial interval was randomly varied between 6 and 9 s. Each condition was presented 10 times in a pseudo-randomized trial order. The threshold for a fast response was adaptive for each subject and each trial to ensure that all subjects were able to win money and work on their maximum performance level. The adaptive algorithm was a simple increase of 5% of the threshold after a slow response and a 10% decrease after a fast response. A detailed description can be found elsewhere (Plichta et al. 2012).

#### Cognitive system—N-back task

The N-back task is a visuo-spatial working memory task, in which numbers from 1 to 4 are visually presented on a screen in a diamond-shaped box (stimulus presentation time: 500 ms, inter-stimulus interval: 1500 ms). There are two conditions presented in a block design: in the 2-back working memory condition, subjects have to encode the current number and simultaneously recall the number seen two trials previously by indicating the spatial location using an MRI compatible diamond-shaped button box. In the 0-back control condition, the subject´s task is to press the button corresponding to the position of the present number. Each block lasted 28 s and both conditions were presented in 4 blocks each. A detailed description can be found elsewhere (Charlet et al. 2014).

#### System for social processes (1)—Faces task

The Faces task is a modified cue-comparison paradigm, according to Hariri et al. (2000) to assess implicit emotion processing. Three facial expressions are presented in a block design (duration: 30 s): one target face in the upper row and two faces in the lower row. Subjects were instructed to respond as fast as possible via button press which of the faces in the lower row matches the target face. Within each block, the presentation of angry and fearful faces was intermixed. As a control condition, geometrical shapes were presented. Each block was repeated four times. A detailed description can be found elsewhere (Wackerhagen et al. 2017).

#### System for social processes (2)—Theory of mind task

The task consists of two alternating conditions: a mentalizing (Theory of Mind, ToM) and a control (non-mentalizing) condition. Each condition started with an instruction followed by a cartoon story consisting of three consecutive pictures. In the ToM condition, subjects had to judge changes in affective states of the protagonist by pressing one of three buttons indicating whether the protagonist felt better, equal or worse than in the picture shown before. All pictures were free of externally visible signs of characters’ emotions such as facial expressions, so affective states had to be inferred by taking the person’s perspective in the respective stories. In the control condition, subjects were asked to evaluate via button presses whether there were more, less or just as much living beings compared to the picture before. A detailed description can be found elsewhere (Mohnke et al. 2016).

### Experimental paradigm-specific brain regions of interest (ROIs) and the networks of interest (NOIs)

To focus on task relevant brain regions, we extracted spatial coordinates from recently published meta-analyses that used the Activation likelihood estimation (ALE) approach proposed by Eickhoff et al. (2009,2012) for each task separately. For the Reward task, we referred to Sescousse et al. (2013); for the N-back task, we referred to Hill et al. (2014, supplementary Table 3); for the Faces task, we referred to Dricu and Frühholz (2016) and for the Theory of mind task we referred to Mar (2011). The above-mentioned coordinates and their anatomical descriptions are given in Supplementary Tables 1–4 in corresponding order. Based on the separate voxel-wise whole-brain analyses described in more detail below, brain responses within paradigm-specific, a priori defined brain regions of interest (ROIs) were examined in more detail. Our so-called Networks of Interest (NOIs) consisted of these ROIs (representing the nodes of the network) and the functional connectivity between these ROIs (representing the edges of the network) which were also the subject of our analyses.

### Brain imaging

#### Data acquisition

Brain scans were acquired on a Siemens Trio 3 T MR scanner at the Charité—Universitätsmedizin Berlin. For functional magnetic resonance imaging (fMRI), we used a blood oxygenation dependent (BOLD) sensitive, T2*-weighted, gradient-echo echo planar imaging (GE-EPI) pulse sequence covering the entire brain (28 slices, descending slice acquisition order, time to repetition = 2 s, time to echo = 30 ms, flip angle = 80°, matrix = 64 × 64, voxel size = 3 × 3 × 4 mm^3^, number of whole head scans: Reward task = 269, N-back task = 135, Faces task = 135, Theory of mind task = 239). In addition, a structural scan with an isotropic resolution of 1 mm^3^ (magnetization prepared rapid acquisition gradient-echo, MPRAGE) was acquired for anatomical reference and display purposes.

#### Image processing

Brain images were analysed using Statistical Parametric Mapping (SPM12, Welcome Trust Centre for Neuroimaging). Images of all tasks were corrected for acquisition delay and head motion and a mean image was computed. The structural scan was co-registered to this mean image and by means of the unified segmentation approach as implemented in SPM12 (Ashburner and Friston 2005) segmented into tissue classes and warped into the reference space defined by the brain template from the International Consortium of Brain Mapping (ICBM). Using the affine and non-linear transformation parameter estimates from this step, functional brain images were also warped, resampled into images with an isotropic voxel size of 3 × 3 × 3 mm^3^ and spatially low pass filtered with an isotropic Gaussian filter of 8 mm full width at half maximum (FWHM). To address low frequency signal fluctuations, voxel time series were finally high pass filtered (cut-off frequency 1/128 Hz) and high frequency and/or aliased respiratory and cardiological noise was removed by means of autoregressive modelling.

#### Modelling of local paradigm-associated brain response

Statistical analyses were conducted within the framework of the Generalized Linear Model (GLM) as implemented in SPM12. First, individual paradigm-associated brain responses were modelled for patient and control persons. To this end, all models consisted of paradigm-specific regressors of interest (detailed explanation see below), the six head motion parameters (to address signal fluctuations caused by susceptibility by motion interactions) and a constant term, modelling the mean of the BOLD time series. Due to the block-wise stimulation characteristics of all paradigms, neural activity was modelled with boxcar functions of corresponding temporal start point and duration. Haemodynamic predictors were then computed by convolution of the neural model with the canonical haemodynamic response function used in SPM. This model was fitted voxel-wise to the data using a restricted maximum likelihood algorithm. Based on the resulting parameter estimates, the following linear contrasts were computed: Reward task: ‘gain anticipation > no gain anticipation’; N-back task: ‘2-back > 0-back’; Faces task: ‘faces > shapes’; ToM task: ‘ToM > control stories’.

#### Modelling of local condition associated brain connectivity

PPI analysis captures the modulation of functional connectivity between brain structures as a function of an experimental or psychological context (Friston et al. 1997; Gitelman et al. 2003). To investigate condition-specific modulation of functional connectivity (FC) within our NOIs, we employed the generalized psycho-physiological interaction approach (gPPI) (McLaren et al. 2012; Friston et al. 1997) using our ROIs (network nodes) as seed regions and calculated for each ROI a voxel-wise gPPI connectivity map. The edges of our NOI were than defined by extracting the $\left({N}_{ROI}^{2}-{N}_{ROI}\right)/2$ mean connectivity values between the network nodes from these maps.

#### Comparison between healthy control group and ASD patient

Based on the contrast images of interest (as taken from single subject brain response and connectivity analyses), we compared the brain responses of the patient with the brain responses of the control group voxel-wise using the method proposed by (Crawford et al. 2009):

1 $${T}_{\left({N}_{HC}-1\right)}=\frac{{x}_{P}-{\overline{x} }_{HC})}{\left(\frac{{SD}_{HC} \sqrt{{N}_{HC}+1}}{{N}_{HC}}\right)}$$

In this formula, *x*_*P*_ is the GLM parameter estimate for the patient in a voxel; ${\overline{x} }_{HC}$ the average of the parameter estimates for the healthy control group, *SD*_*HC*_ its corresponding standard deviation and *N*_*HC*_ the sample size of this group. The computation yields a *t*-value with N_HC_−1 degrees of freedom. To statistically compare socio-demographical, psychometrical and clinical data between patient and control group, we used the same method.

#### Alpha error correction for multiple comparisons

The resulting statistical brain maps of the patient were thresholded with a conventional statistical threshold of *p* < 0.05 (uncorrected for multiple comparisons). Based on the field smoothness estimates provided by SPM, we conducted in a second step a Monte Carlo simulation to estimate the probability of a given cluster size to occur for the above-mentioned uncorrected threshold. For this step, we used the software AlphaSim as implemented in the REST toolbox for SPM (Song et al. 2011). The underlying field smoothness estimates as well as the resulting minimum cluster sizes for a corrected significance level of *p* < 0.05 all analyses are given in Supplementary Table 5.

The brain response within a certain ROI was only considered for report and discussion if a cluster of above threshold size, as revealed by the corresponding voxel-wise whole-brain analysis, overlapped with this ROI. In gPPI analysis, this overlap criterion was applied to all non-seed ROIs to identify significant connections.

## Results

### Psychometrics

In neuropsychological tests and personality ratings, the case patient differed significantly from the control group regarding years of education but not in highest school degree or premorbid intelligence as assessed by the multiple-choice verbal intelligence test (MWT-B, Lehrl 2005) or fluid intelligence as assessed by the matrices test (HAWIE-R, Tewes 1991). A comparison of personality scales revealed a distinction between the case patient and the control group in the NEO Five-Factor Inventory (NEO-FFI, Costa and McCrae 1992) as well as the Temperament and Character Inventory (TCI, Cloninger 1994) (see Table 1).

**Table 1:** Psychometrics

	Patient with ASD (1 ♀)	HC (14 ♀)		ASD > HC *T*_13_ (*p*)
		Mean	SD	
Demographics				
Age in years	48	45	2.43	1.05 (.157)
Years of education	21	14	1.73	3.61 (.002)
Highest degree of education	7	6	0.93	1.36 (.098)
IQ				
Multiple choice verbal intelligence test	30	31	2.68	− 0.48 (.321)
Matrices test	23	18	3.29	1.44 (.087)
NEO-FFI				
Neuroticism	33	12	7.51	2.65 (.010)
Extraversion	3	33	4.15	− 7.79 (< .001)
Openness to experience	16	31	6.40	− 2.15 (.026)
Agreeableness	20	38	3.5	− 4.70 (< .001)
Conscientiousness	36	39	4.62	− 0.53 (.302)
TCI				
Harm avoidance	26	11	6.49	2.18 (.024)
Novelty seeking	4	22	3.69	− 4.48 (< .001)
Reward dependence	5	17	2.0	− 5.58 (< .001)

*ASD* autism spectrum disorder, *HC* healthy control group, *SD* standard deviation, *IQ* intelligence quotient, *NEO-FFI* NEO Five-Factor Inventory, *TCI* Temperament and Character Inventory. Levels of education: 1 = primary education without certificate; 2 = primary education certificate; 3 = lower secondary education without certificate; 4 = lower secondary education certificate; 5 = secondary education certificate; 6 = entrance qualification for studying at a university of applied sciences; 7 = general higher education entrance qualification

### Performance in experimental paradigms

In the N-back task, the case patient did not significantly differ from the control group on a behavioural level. Regarding her own motivation for the N-back task, our patient reported: “Since I assumed that my test performance on the social processing task would not be good, while performance on the Reward task may be just average, and because I knew that I would learn about my “ranking” with respect to test performance afterwards, it was very important for me to have a very good result in this task.”

The gain achieved by the patient in the Reward task was significantly higher than the mean gain in the control group (16 vs. 7 €). As the interest in monetary rewards may differ between persons with ASD and control subjects (Kohls et al. 2013), we inquired about the motivational value in the reward anticipation task and our patient stated: “Monetary reward means nothing to me because I do not know what to do with it. This was already the case when I was a child: I cannot remember that I ever bought myself anything with my pocket money with the exception of a bicycle after 10 years. I guess that this is because the “small wishes” that can be fulfilled with such a reward are always associated with social interaction (shopping, being in a café etc.). In this context, the anxiety to be rejected or humiliated outweighs the potential “utility” of shopping. That my performance did not decline more may be due to the fact that I decided to donate the money that I gained, which motivated me to do well.”

In the Faces task, the case patient needed significantly longer to match faces (*T*(13) = 5.94; *p* < 0.001). This was not the case for the forms matching condition (see Table 2). As persons with ASD may display prosopagnosia, we inquired and our patient reported “[This task was] extremely difficult for me: what I—as far as I noticed—intuitively do is to focus on items (hair colour, the way the hair is cut, the form of the face etc.), that are not helpful. Instead, I had to focus on the form of the eyes and/or the mouth, and comparing these aspects takes a lot of time. I feel that this is not really a “social processing task”, because all you have to do is to compare pixels with each other.”

**Table 2:** Performance during MRI-experiment

	Patient with ASD (1 ♀)	HC (14 ♀)		ASD > HC T_13_ (*p*)
		Mean	SD	
Positive valence system—Reward task				
Total gain (€)	16.00	7.29	4.55	1.85 (.043)
Win, RT (s)	1.87	2.47	0.50	− 1.12 (.141)
Loss, RT (s)	1.72	2.52	1.23	− 0.63 (.270)
Cognitive system—N-back task				
0back, correct (%)	100.00	96.81	5.65	0.54 (.299)
2back, correct (%)	72.92	59.97	20.00	0.63 (.270)
0back, RT (s)	0.54	0.60	0.07	− 0.85 (.205)
2back, RT (s)	0.72	0.76	0.28	− 0.13 (.451)
System for social processes (1)—Faces task				
Faces, correct (%)	100.00	98	2.23	0.84 (.208)
Faces, RT (s)	1.90	1.23	0.11	5.74 (< .001)
Forms, RT (s)	1.05	1.12	0.15	− 0.42 (.342)
System for social processes (2)—Theory of mind task				
Social cognition, correct (%)	56.25	52.23	14.42	0.19 (.425)
Social cognition, RT (s)	4.10	3.41	0.74	0.90 (.193)

*ASD* autism spectrum disorder, *HC* healthy control group, *SD* standard deviation, *RT* response time

In the ToM task, the case patient did not differ significantly from controls. Qualitatively, our patient explained her task strategy: “Difficult for me was not the real Theory of Mind part but rather the decoding of the comic strips. I generally have these problems with comics. I think this is because “typical” features are exaggerated to illustrate to the reader what is intended. However, when the intended meaning depends upon decoding such an exaggerated feature, I do not get the story. I have the same problem with political caricatures.” These explanations point to the difficulties that our patient already described when having to decode affect from facial expressions in the Faces task.

We would like to emphasize the limitation that this study is a case report of one female participant compared with other females and refer to the discussion for a more detailed presentation of the limitations of this approach.

### Brain imaging

Loud noise can be a substantial stress factor for patients with ASD, thus impairing their performance (Robertson and Baron-Cohen 2017). Regarding scanner noise, our patient reported**:** “I felt well protected by having ear plugs against the noise and being promised that nobody would be in the room, so for example I would not be confronted with unexpected sensations. Imagining that somebody may have come in and (even well intended) touch my shoulder to encourage me, or accidently bump into me or that I would not have earplugs and be confronted with sudden noises would all have been horrible. It may have made me feel nauseated. In any case, would have needed several hours of absolute tranquility to recover. Since this was not the case and I was relaxed, doing the tests was fun.” In general, the results of the brain imaging paradigms have to be treated with caution, as we only rely on one participant.

#### Positive valence system—Reward task

##### Brain response—whole-brain analysis

For the contrast ‘gain anticipation > no gain anticipation’ the patient with ASD showed reduced brain responses in several, mostly cortical brain regions. Most pronounced effects were found in bilateral insula lobes. Along the mesolimbic dopaminergic pathway, the patient P.K. did not differ from the control group regarding reward responses in the ventral striatum (VS). For a detailed depiction of the results please see Supplementary Table 6 and Supplementary Fig. 1.

##### Brain response—network analysis according to Sescousse et al. (2013)

Three out of the 21 brain regions that are defined as network nodes in network analysis showed reduced brain response on rewarding stimuli: the right posterior cingulate cortex and the right ventrolateral as well as dorso-medial thalamus. Beyond this, several midline and frontal structures fell short of significance, yet pointing to a decreased brain response. The only trend towards an increased brain response was located in left-hemispheric visual cortex.

##### Brain connectivity (gPPI)—network analysis according to Sescousse et al. (2013)

**Fig. 1:**
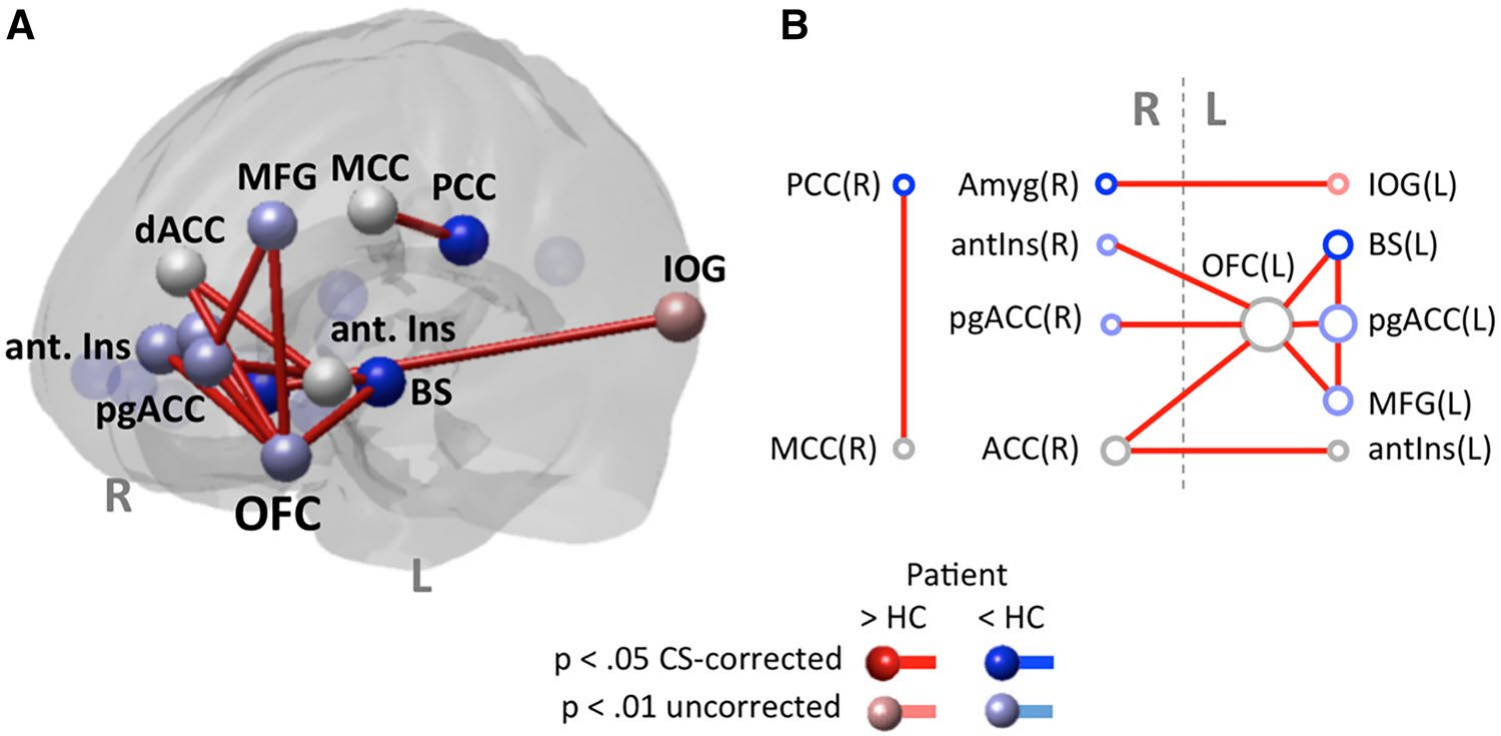
Reward task—results of network analysis. Results of network analyses on differences in brain response and functional connectivity (gPPI) according to Table 3 displayed in brain space (**a**) and as connectivity graph (**b**). Nodes with elevated response are shown in red; those with reduced response are shown in blue. In case of comparable brain response, nodes are displayed in light grey. Findings passing *p* < .05 cluster size correction for multiple comparisons are displayed in full shade, those not correctable are displayed in pastel shade. Node names/brain regions according to Table 3,* L* left, *R* right, *Pat* patient, *HC* healthy control group, *CS-corrected* cluster size corrected

In contrast to the generally reduced brain response, we found pronounced modulation of intra-frontal connectivity in the patient with ASD. Notably, the orbitofrontal ROI was involved in 5 out of altogether 10 between-ROI connections and was more affected by experimental condition in the patient with ASD than in HCs. The findings of brain response and connectivity network analysis are listed in Table 3 and displayed in Fig. 1.

**Table 3:** Reward task—results of network analysis

Node/brain region	H	T(13)	*p*	Node degree
Posterior cingulate cortex (PCC)	R	− 4.95	< .001*	1*
Ventromedial prefrontal cortex (VLPFC)	R	− 4.44	.001*	0
Brainstem (BS)	L	− 4.23	< .001*	2*
Ventromedial prefrontal cortex (VLPFC)	L	− 3.21	.003*	0
Amygdala	R	− 2.91	.006*	1*
Ventral striatum (VS)	L	− 2.84	.007*	0
Thalamus (MD)	R	− 2.68	.009*	0
Posterior orbitofrontal cortex (OFC)	L	− 2.54	.012	6*
Perigenual anterior cingulate cortex (pgACC)	L	− 2.56	.012	3*
Middle frontal gyrus (MFG)	L	− 2.04	.031	2*
Dorsal anterior cingulate cortex (dACC)	R	− 1.17	.131	2*
Inferior occipital gyrus (IOG)	L	3.81	.001	1*
Anterior insula/inferior frontal gyrus (IFG)	R	− 2.27	.020	1*
Perigenual anterior cingulate cortex (pgACC)	R	− 2.19	.024	1*
Insula (anterior)/IFG	L	− 1.75	.054	1*
Middle cingulate cortex (MCC)	R	− 0.97	.175	1*

A priori defined brain regions that represent nodes of the NOIs with significant differences in brain response or brain connectivity (gPPI) between ASD and HC. The degree of a node equals the number of other nodes this node is connected with. All connections are *p* < .05 cluster size corrected for multiple comparisons. Asterisks highlight error adjustment for multiple comparisons in brain responses explicitly

*H* hemisphere, *L* left, *R* right

#### Cognitive system—N-back task

##### Brain response—whole-brain analysis

Compared with the healthy control group, the patient with ASD showed a widespread increase in brain response on working memory demands (2-back > 0-back). Elevated brain responses were observed in bilateral basal ganglia including the ventral striatum and thalamus, the left insula, bilateral premotor regions on middle and superior frontal gyrus and the right visual cortex. In contrast, reductions in neural activity were restricted to bilateral cerebellar and right hemispherical inferior temporal structures. For a detailed depiction of the results please see Supplementary Tables 7 and 8 as well as Supplementary Fig. 2.

##### Brain response—network analysis according to Hill et al. (2014)

Two out of the ten network nodes showed elevated brain responses in the patient with ASD: the bilateral middle frontal gyri comprising Brodmann areas 6 and 9. A trend towards increased activity was also found in bilateral parietal regions, the right prefrontal cortex, the cingulate cortex and the precuneus. A decrease in brain responses within the network could not be detected.

##### Brain connectivity (gPPI)—network analysis according to Hill et al. (2014)

**Fig. 2:**
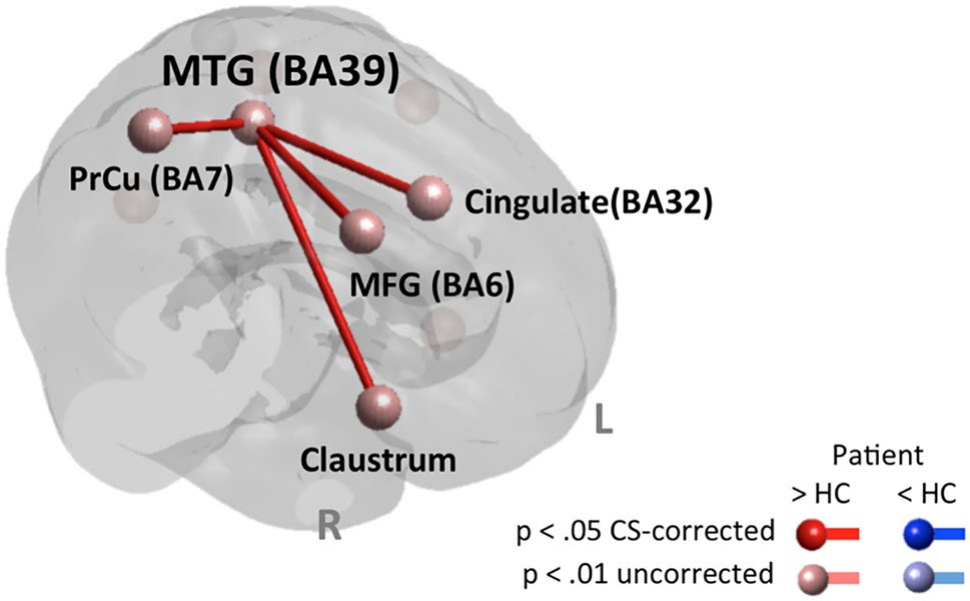
Cognitive system—N-back task. Results of network analyses on differences in brain response and functional connectivity (gPPI) according to Table 4. Nodes with elevated response are shown in red; those with reduced response are shown in blue. In case of comparable brain response, nodes are displayed in light grey. Findings passing a *p* < .05 cluster size corrected for multiple comparisons are displayed in full shade, those not correctable are displayed in pastel shade. Node names/brain regions according to Table 3,* L* left, *R* right, *Pat* patient, *HC* healthy control group, *CS-corrected* cluster size corrected

Compared to the healthy control group, long-distance fronto-parietal functional connectivity was more intensely modulated in the patient with ASD. Interestingly, the ROI for BA39 (a part of the TPJ) was always involved. The findings of brain response and connectivity network analysis are listed in Table 4 and displayed in Fig. 2.

**Table 4:** Cognitive system—N-back task

Node/brain region	H	T(13)	*p*	Node degree
Claustrum	R	2.74	.008	1
Middle Frontal Gyrus (MFG, BA 6)	R	2.45	.015	1
Precuneus (BA 7)	R	2.22	.022	1
Middle Temporal Gyrus (MTG, BA 39)	L	2.08	.029	4
Cingulate (BA 32)	L	1.92	.039	1

A priori defined brain regions that represent nodes of the NOIs with significant differences in brain response or brain connectivity (gPPI) between ASD and HC. The degree of a node equals the number of other nodes this node is connected to. All connections are *p* < .05 cluster size corrected for multiple comparisons. Asterisks highlight error adjustment for multiple comparisons in brain responses explicitly

*H* hemisphere, *L* left, *R* right

#### System for social processes (1)—Faces task

##### Brain response—whole brain

Elevated brain responses were found in posterior brain regions, specifically in ventral visual areas such as calcarine and lingual gyrus, but also in the left-hemispheric cerebellar lobules VI and VII. For a detailed depiction of the results please see Supplementary Tables 9 and Supplementary Fig. 3.

##### Brain response and connectivity—network analysis according to Dricu and Früholz (2016)

Within this on network, only a trend towards elevated brain responses was found in the right precentral gyrus and left fusiform gyrus.

#### System for social processes (2)—Theory of mind task

##### Brain response

Brain response—whole-brain analysis: compared with our control group, only in precuneus we found a cluster size-correctable reduction in P.K.’s brain responses during social cognition (ToM-stories > control stories).

Brain response—network analysis according to Mar et al. (2011): in network node analysis, the effect regarding a reduction of activity within the precuneus was confirmed. In addition, a trend towards a reduction of brain responses in left AG/STG/TPJ was detected.

Brain connectivity—network analysis according to Mar et al. (2011): no alterations in functional connectivity modulation between network nodes could be found in the patient compared to HCs.

**Fig. 3:**
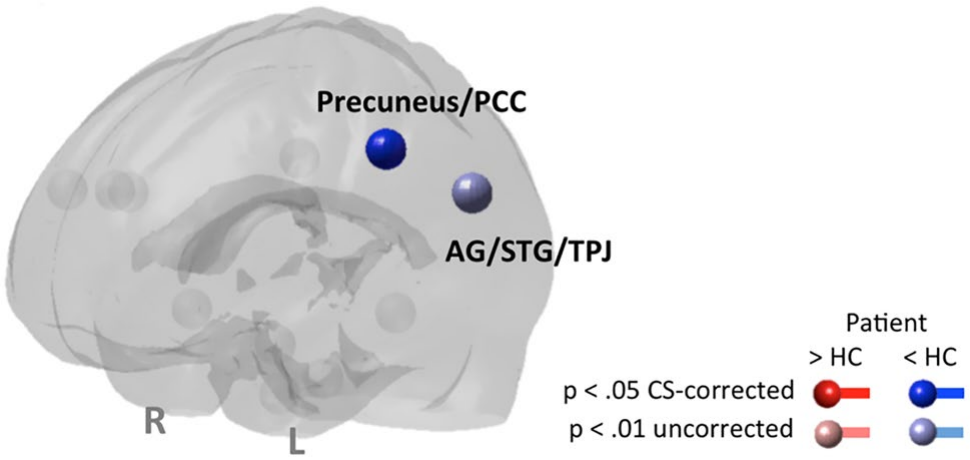
System for social processes (2)—Theory of mind task. Results of network analyses on differences in brain response and functional connectivity (gPPI) according to Table 4. Nodes with elevated response are shown in red; those with reduced response are shown in blue. In case of comparable brain response, nodes are displayed in light grey. Findings passing *p* < .05 cluster size correction for multiple comparisons are displayed in full shade, those not correctable are displayed in pastel shade. Node names/brain regions according to Table 3, *L* left, *R* right, *Pat* patient, *HC* healthy control group, *CS-corrected* cluster size corrected

The findings of brain response and connectivity network analysis are listed in Table 5 and displayed in Fig. 3.

**Table 5:** System for social processes (2)—Theory of mind task

Node/brain region	H	T(13)	*p*	Node degree
Precuneus/posterior cingulate cortex	L	− 4.15	< .001*	0

A priori defined brain regions that represent nodes of the NOIs with significant differences in brain response or brain connectivity (gPPI) between ASD and HC. The degree of a node equals the number of other nodes the node is connected with. All connections are *p* < .05 cluster size corrected for multiple comparisons. Asterisks highlight error adjustment for multiple comparisons in brain responses explicitly

*H* hemisphere, *L* left, *R* right

## Discussion

We compared the rare case of a high-functioning female individual with ASD with a healthy control group matched on gender and age. To this end, we used four RDoC domain specific tasks covering the domains of positive valence, the cognitive system and the system for social processes.

On the behavioural level, the patient showed increased response times during the Faces task and achieved a higher total gain in the Reward task, whereas her performance in N-back and ToM task was similar to HC. This in line with our assumptions, with exception of the average performance in the ToM task. The ability to perform well in the ToM task may reflect the above-average social skills of or patient. Also, the patients report concerning difficulties while decoding of the stimulus content rather than the ToM-associated conclusions strengthen this interpretation.

On the neural level, our findings were somewhat unexpected. In contrast to our assumptions, we observed the most pronounced differences in brain response between patient and healthy controls in the positive valence and cognitive domains, but not in the social processes domain, which is usually reported to be affected in the most common occurrences of ASD (Neuhaus et al. 2010; e.g., Fernández et al. 2018; Kliemann et al. 2018). Interestingly, most of these differences were related to an increase in functional connectivity and a decrease in brain responses in our patient.

The Reward task applied to assess the positive valence system has been studied extensively (for review see e.g., Knutson and Heinz 2015) and reliably evokes strong neural activity in healthy participants along the mesolimbic dopaminergic system specifically within the ventral striatum. However, group differences between patients with ASD and healthy controls for Reward tasks are not consistent (Delmonte et al. 2012; Matyjek et al. 2020a, b). In our reported case no alterations along the mesolimbic pathway were detectable, whereas frontal brain responses to reward announcing cues in target regions of mesocortical pathway, specifically within the bilateral anterior insula, were reduced. This suggests an altered salience attribution or appraisal of monetary incentives of our subject during the task, which is emphasized by her subjective experience of not feeling to benefit from a monetary gain. The observed reduction in posterior cingulate activity in the patient may correspond to less pronounced self-referential processes. This is in line with the patient’s decision to donate the financial gain from this task, which was described as a strategy of hers to reduce possible anxiety related to social interactions while spending money. Cognitively detaching her task performance from the personal relevance or meaning of money may have facilitated an above-average task performance as she applied a performance-focussed rather than reward-focussed strategy. Another explanation for the observed pattern of alterations could be a stronger modulation in the default mode network activity in cortical midline regions or a superposition of both effects (e.g., Assaf et al. 2010), which we assume as less likely. Ultimately, the Reward task may have tapped the cognitive system rather than the positive valence system for our patient.

Surprisingly, our patient displayed significant activation of dopamine-rich brain areas (i.e. ventral striatum) in the N-back task of the cognitive domain, while this had not been the case for the monetary Reward task. Taking the self-report of the patient into account, this activation is not surprising as she described to experience the task as rewarding. In line with our findings, Dichter et al. (2012) observed increased brain activation in response to monetary incentives, when patients were confronted with stimuli that are known to be salient for individuals with ASD (e.g., trains, electronic devices, computers etc.). Here, the reward-related brain circuitry significantly responded to these autism-relevant object images. Since our subject P.K. is an expert in Mathematics, the task of memorizing numbers poses a possibly rewarding situation. An elevation of left-hemispheric insular and inferior frontal brain responses as a part of the so-called salience network (Peters et al. 2016) may point to a higher salience of this task for the patient, which was again confirmed by the patient’s subjective report to be “very motivated to achieve good results”. On the other hand, more intense or different rehearsal processes may evoke stronger brain responses in these partially language-associated brain regions (e.g., Hagoort and Indefrey 2014). Again, taking the subjective report of the patient into consideration, the N-back task here may have also targeted the positive valence dimension rather than the cognitive dimension alone.

Within the in ASD usually strongly affected domain of social processes, the brain responses of patient P.K. on both experimental tasks did not differ significantly from those of the control group. Considering the heterogeneity regarding social cognition in subjects with ASD, our patient seems to range in the more functional part of the spectrum in that respect (Baron-Cohen et al. 2015; Kliemann et al. 2018), which her extraordinary academic achievements may reflect. However, the observed brain response and connectivity pattern as well as the significantly higher response times for the face matching condition point at an increased effort of P.K. in solving this task: (1) The slight increase in cortical activation along the ventral visual stream, and (2) the slight increase in TPJ activity as well as a simultaneous slight decrease in posterior cingulate/precuneus brain response during the comic-based ToM task. Specifically, the latter mentioned reduced response on ToM-associated stimuli, might indicate a lowered integration of emotional content within the cartoon stories in the service of spatial orientation and memory (Vogt et al. 1992) and lowered visuo-spatial imagery and self-referential operations during this task in the patient (e.g., Cavanna and Trimble 2006). This interpretation is emphasised by her report of not experiencing this task as a “social processing” task, but rather a perceptual “decoding” task.

However, there are several issues to be considered when interpreting our data. First, given the case report approach, caution is warranted when seeking to generalize the results to the wider population of individuals with ASD. Secondly, because our highly functional patient had task goals and solution strategies that differed from those of healthy controls or even other ASD patients, the feasibility of the used tasks could be questioned in our case. To address this issue, we considered patient reports while interpreting the results of our studies.

Thirdly, we are focussing on a female patient with ASD. There is evidence for differences in cognition (e.g., Hull et al. 2017b) as well as brain structure and function (Lai et al. 2017 for review) between females and males with ASD. Interestingly, a recent study showed differences in brain connectivity between females and males with ASD, with females displaying higher connectivity in a range of brain networks (Alaerts et al. 2016; Smith et al. 2019), whereas for patients with ASD compared to healthy individuals’ results are heterogenous with both evidence for increased and reduced connectivity (Hull et al. 2017a; O’Reilly et al. 2017). Our results yielded more pronounced connectivity in the female patient with ASD in the reward and memory tasks compared to the female control group, which might be a reflection of our patient's exceptional functional level, which allowed a high academic career. Moreover, gender is very likely a general resilience factor in ASD (Szatmari 2018). Thus, also some of our findings may be of more general nature and not solely unique to our case.

Finally, to increase generalizability of our findings and for a better understanding of compensatory mechanisms in ASD, future studies should compare groups of individuals with ASD and occupational success with ASD controls without occupational success, matched on education and IQ. That being said, focussing on educational success as done in the current study is but one possible approach to elucidate compensatory mechanisms in ASD. Given that such success depends not only on intraindividual factors of compensation but also on a person-environmental fit (Lai et al. 2020), variability is needed in such study designs, which demands high sample sizes. Other design approaches such as focussing on individuals with preserved functioning in areas that are usually affected in ASD, as realized with e.g., preserved Theory of mind function (White et al. 2014), should therefore also be exploited further (cf. Livingston and Happé 2017).

## Conclusions

Our study yielded an unexpected pattern of differences and similarities in brain activity between our high-functioning female patient with ASD and a matched female control sample and shows that the value added of reported subjective experiences can be high in clinical case reports, as it reveals unexpected task strategies and achievement motives of the patient and therefore provides an extended framework of interpretation for brain activity in the experimental paradigms. Given the reliance on one patient though, our findings should be treated with caution. However, due to their sparse appearance and high inter-individual variability, the value of case studies should not be underestimated, as a systematic investigation of such cases on group level is difficult or even impossible.

Our observations provide additional information on the neural basis of the high-functional level of our patient: in our opinion, it is likely that an adaptation of P.K.s task-related brain connectivity contributes more to her high-functional level than a more intense cortical information processing (i.e., increased brain response). Perhaps adaptive capabilities and their neurobiological correlates including high levels of functional connectivity enable P.K. to overcome her ASD-related constraints and to be professionally successful above average, emphasizing the variety of neurobiological underpinnings of specific diagnosis and their individual functional level.

## Supplementary Information

Below is the link to the electronic supplementary material.

Supplementary file1 (DOCX 1347 kb)

## Data Availability

The datasets used and/or analysed during the current study are available from the corresponding author on reasonable request.

## Code availability

The code used in the current study are available from the corresponding author on reasonable request. The software used during the current study is available.
