# Supplementary material for: Neural correlates of RDoC-specific cognitive processes in a high-functional autistic patient: a statistically validated case report
Source: J Neural Transm (Vienna). 2021 May 18;128(6):845–59. doi: 10.1007/s00702-021-02352-w (PMC8205905; doi:10.1007/s00702-021-02352-w)
Supplement: Supplementary file 1 — Supplementary file1 (DOCX 1347 kb) [file 702_2021_2352_MOESM1_ESM.docx]

Supplementary Material

**Neural correlates of RDoC specific cognitive processes in a high-functional autistic patient:**

**a statistically validated case report.**

Laura S Daedelow^1^

Anne Beck, PhD^1^

Lydia Romund, PhD^1^

Lea Mascarell-Maricic^1^

Isabel Dziobek, PhD^2,3^

Nina Romanczuk-Seiferth, PhD^1^

Torsten Wüstenberg*, PhD^1,4^

Andreas Heinz*, MD, PhD^1^

^1^ Department of Psychiatry and Psychotherapy, Charité - Universitätsmedizin Berlin, Charité Campus Mitte, Berlin, Germany

^2^ Berlin School of Mind and Brain, Berlin, Germany

^3^ Department of Psychology, Humboldt-University of Berlin, Berlin, Germany

^4^ Systems Neuroscience in Psychiatry (SNiP), Central Institute of Mental Health, Mannheim, Germany

^*^These authors contributed equally to this work.

Reward: MID task

**Supplementary Table 1: Reward network-nodes** according to Sescousse et al. (2013).

| **#** | **Brain region** | **Coord. (mm)** | | | **Ref.- space** | **Description** |
| --- | --- | --- | --- | --- | --- | --- |
|  |  | **x** | **y** | **z** |  |  |
|  |  |  |  |  |  |  |
|  |  |  |  |  |  |  |
| 1 | VS | -14 | 10 | -12 | MNI | Ventral striatum (nucleus accumbens) |
| 2 | VS | 14 | 10 | -8 | MNI | Ventral striatum (nucleus accumbens) |
| 3 | Amygdala | 24 | -2 | -18 | MNI | Amygdala |
| 4 | Thalamus (MD) | 4 | -12 | 10 | MNI | Mediodorsal thalamus |
| 5 | Thalamus (VL) | 22 | -24 | -8 | MNI | Posterior ventrolateral thalamus |
| 6 | Insula (anterior)/IFG | -28 | 16 | 0 | MNI | Anterior insula / Frontal operculum |
| 7 | Insula (anterior)/IFG | 36 | 22 | -6 | MNI | Anterior insula / Frontal operculum |
| 8 | pgACC | -12 | 42 | 6 | MNI | Perigenual ACC |
| 9 | pgACC | 2 | 34 | 8 | MNI | Perigenual ACC |
| 10 | ACC | 8 | 36 | 24 | MNI | ACC |
| 11 | VMPFC | 0 | 44 | -8 | MNI | Ventromedial PFC (superior frontal gyrus) |
| 12 | VMPFC | 0 | 58 | 0 | MNI | Ventromedial PFC (superior frontal gyrus) |
| 13 | VMPFC | 12 | 60 | 0 | MNI | Ventromedial PFC (superior frontal gyrus) |
| 14 | Midbrain | -4 | -18 | -12 | MNI | Midbrain |
| 15 | OFC (posterior) | -32 | 32 | -16 | MNI | Posterior orbitofrontal cortex |
| 16 | MFG | -26 | 32 | 44 | MNI | Middle frontal gyrus |
| 17 | PCC | -2 | -34 | 30 | MNI | Posterior cingulate cortex |
| 18 | PCC | 4 | -50 | 18 | MNI | Posterior cingulate cortex |
| 19 | IOG | -40 | -86 | 2 | MNI | Inferior occipital gyrus |
| 20 | PCC | 2 | -20 | 32 | MNI | Posterior cingulate cortex |
| 21 | Cuneus/LG | 12 | -86 | 4 | MNI | Cuneus / Lingual gyrus R |
|  |  |  |  |  |  |  |

***Abbreviations:*** *MNI – Montreal Neurological Institute reference space, ACC – Anterior Cingulate Cortex, PFC – Prefrontal Cortex.*

Working memory: N-back task

**Supplementary Table 2: Working memory network**-nodes according to Hill et al. (2014).

| **#** | **Brain region** | **Coord. (mm)** | | | **Ref.-space** | **Description** |
| --- | --- | --- | --- | --- | --- | --- |
|  |  | **x** | **y** | **z** |  |  |
|  |  |  |  |  |  |  |
|  |  |  |  |  |  |  |
| 1 | MFG (BA 6) | 26 | 2 | 52 | TAL | Middle Frontal Gyrus (BA 6) |
| 2 | MFG (BA 9) | 28 | 28 | 30 | TAL | Middle Frontal Gyrus (BA 9) |
| 3 | MFG (BA 6/9) | -28 | -4 | 50 | TAL | Middle Frontal Gyrus (BA 6/9) |
| 4 | Cingulate (BA 32) | -4 | 10 | 42 | TAL | Cingulate Gyrus (BA 32) |
| 5 | Precuneus (BA 7) | 12 | -64 | 48 | TAL | Precuneus (BA7) |
| 6 | Precuneus (BA 19) | 30 | -60 | 40 | TAL | Precuneus (BA 19) |
| 7 | SPL (BA 7) | -28 | -62 | 48 | TAL | Superior Parietal Lobule (BA 7) |
| 8 | IPL (BA 40) | -34 | -50 | 36 | TAL | Inferior Parietal Lobe (BA 40) |
| 9 | Claustrum | 32 | 14 | 0 | TAL | Claustrum |
| 10 | MTG (BA 39) | -32 | -50 | 20 | TAL | Middle Temporal Gyrus (BA 39) |
|  |  |  |  |  |  |  |

***Abbreviations:*** *TAL – Talairach reference space, BA – Brodmann area.*

Face perception: Face-matching task

**Supplementary Table 3: Face perception network-nodes** according to Dricu et al. (2016).

| **#** | **Brain region** | **Coord. (mm)** | | | **Ref.-space** | **Description** |
| --- | --- | --- | --- | --- | --- | --- |
|  |  | **x** | **y** | **z** |  |  |
|  |  |  |  |  |  |  |
|  |  |  |  |  |  |  |
| 1 | Amygdala | -22 | -8 | -18 | MNI | Amygdala |
| 2 | FG (middle) | -42 | -52 | -20 | MNI | Middle fusiform gyrus |
| 3 | IOG | 46 | -76 | -6 | MNI | Inferior occipital gyrus |
| 4 | PCG | 54 | 8 | 38 | MNI | Precentral gyrus |
| 5 | Amygdala | 22 | -12 | -16 | MNI | Amygdala |
| 6 | STG (posterior) | 60 | -44 | 4 | MNI | Posterior superior temporal sulcus |
|  |  |  |  |  |  |  |

***Abbreviations:*** *MNI – Montreal Neurological Institute reference.*

Theory of mind: Comic story task

**Supplementary Table 4: Theory of Mind (ToM) network-nodes** according to Mar et al. (2011).

|  | **Brain region** | **Coord. (mm)** | | | **Ref.- space** | **Description** | |
| --- | --- | --- | --- | --- | --- | --- | --- |
| **#** |  | **x** | **y** | **z** |  |  |  |
|  |  |  |  |  |  |  | |
|  |  |  |  |  |  |  | |
| 1 | MPFC/ACC | 4 | 58 | 28 | MNI | Medial prefrontal cortex: frontal pole & medial superior frontal gyrus, anterior cingulate cortex | |
| 2 | ACC | 18 | 28 | 24 | MNI | Anterior cingulate cortex | |
| 3 | Precuneus/PCC | -10 | -50 | 36 | MNI | Precuneus/posterior cingulate cortex | |
| 4 | AG/STG/TPJ | -52 | -58 | 26 | MNI | Angular gyrus/superior temporal sulcus/TPJ, posterior superior temporal gyrus, posterior middle temporal gyrus | |
| 5 | AG/STG/TPJ | 54 | -54 | 26 | MNI | Angular gyrus/superior temporal sulcus/TPJ, posterior superior temporal gyrus | |
| 6 | MTG | -56 | -28 | -10 | MNI | Middle temporal gyrus/superior temporal sulcus | |
| 7 | MTG | 56 | -16 | -20 | MNI | Middle temporal gyrus/superior temporal sulcus | |
| 8 | Temporal pole | -52 | 12 | -34 | MNI | Temporal pole | |
| 9 | SFG | -20 | 54 | 30 | MNI | Superior frontal gyrus | |
| 10 | Amygdala/PHG | -24 | -2 | -22 | MNI | Amygdala/parahippocampal gyrus | |
| 11 | MTG (anterior) | -52 | -4 | -30 | MNI | Anterior middle temporal gyrus, inferior temporal gyrus | |
|  |  |  |  |  |  | |  |

***Abbreviations:*** *MNI – Montreal Neurological Institute reference space.*

**Supplementary Table 5:** Alpha error adjustment: Cluster size estimates as computed by means of 1000 Monte-Carlo simulations for a corrected p<.05 for the given smoothness of SPMs and in initial uncorrected p-value of .001.

| **Task** | **FWHM (mm)** | | | **Minimal CS (voxels)** |
| --- | --- | --- | --- | --- |
| Brain response | **X** | **Y** | **Z** |  |
| gPPI: seed |  |  |  |  |
| **Reward** |  |  |  |  |
| Brain response | 13.2 | 13.5 | 13.3 | 65 |
| gPPI: left posterior Orbitofrontal Cortex – **OFC (L)** | 10.7 | 11.1 | 10.3 | 44 |
| gPPI: left anterior Insula – **Ins (L)** | 11.4 | 11.1 | 10.8 | 46 |
| gPPI: left Middle Frontal Gyrus – **MFG (L)** | 12.9 | 13.9 | 13.3 | 66 |
| gPPI: left perigenual Anterior Cingulate Cortex – **pgACC (L)** | 11.7 | 12.1 | 11.6 | 54 |
| gPPI: left Midbrain/Brainstem – **BS (L)** | 12.0 | 11.8 | 11.4 | 54 |
| gPPI: left Posterior Cingulate Cortex – **PCC (L)** | 10.6 | 10.9 | 10.3 | 43 |
| gPPI: left Ventromedial Prefrontal Cortex – **VMPFC (L**) | 15.8 | 16.1 | 15.7 | 84 |
| gPPI: right perigenual Anterior Cingulate Cortex – **pgACC (R)** | 11.5 | 11.7 | 11.7 | 50 |
| gPPI: right Posterior Cingulate Cortex 1st – **PCC1st (R)** | 11.5 | 11.7 | 10.8 | 48 |
| gPPI: right mediodorsal Thalamus – **Thal (R)** | 11.8 | 12.2 | 11.0 | 53 |
| gPPI: right Posterior Cingulate Cortex 2nd – **PCC2nd (R)** | 11.0 | 11.2 | 10.5 | 45 |
| gPPI: right Anterior Cingulate Cortex – **ACC(R)** | 14.9 | 15.8 | 15.4 | 80 |
| gPPI: right Ventromedial Prefrontal Cortex – **VMPFC (R)** | 10.9 | 11.1 | 10.8 | 43 |
| gPPI: right Cuneus/Lingual gyrus – **Cu/LG (R)** | 12.1 | 12.2 | 11.8 | 52 |
| gPPI: right Ventral Striatum – **VS (R)** | 12.3 | 12.4 | 12.2 | 56 |
| gPPI: right posterior ventral Thalamus – **Thal (R)** | 10.6 | 10.7 | 10.3 | 43 |
| gPPI: right Amygdala – **Amyg (R)** | 13.4 | 13.0 | 13.2 | 65 |
| gPPI: right anterior Insula - **Ins (R)** | 11.2 | 11.5 | 11.2 | 45 |
| **N-back** |  |  |  |  |
| Brain response | 13.9 | 13.9 | 13.0 | 72 |
| PPI: IPL/BA40(L) | 12.1 | 12.3 | 11.7 | 54 |
| PPI: MTG/BA39(L) | 12.5 | 13.2 | 11.9 | 59 |
| PPI: SPL/BA7(L) | 12.9 | 12.6 | 11.9 | 54 |
| PPI: Cingulate/BA32(L) | 12.5 | 12.3 | 11.9 | 57 |
| PPI: PrCu/BA7(R) | 13.0 | 12.8 | 12.1 | 55 |
| PPI: MFG/BA6(R) | 11.9 | 12.1 | 11.4 | 52 |
| PPI: MFG/BA9(R) | 11.5 | 11.4 | 11.1 | 49 |
| PPI: PrCu/BA19(R) | 12.0 | 12.1 | 11.5 | 53 |
| PPI: Claustrum(R) | 13.5 | 13.4 | 12.9 | 67 |
| **Faces** |  |  |  |  |
| Brain response | 13.9 | 13.0 | 13.6 | 69 |
| PPI: FG(L) | 11.9 | 11.9 | 11.4 | 50 |
| PPI: Amyg (L) | 11.3 | 11.3 | 11.3 | 48 |
| PPI: Amyg (R) | 11.1 | 11.2 | 10.9 | 47 |
| PPI: post. STS(R) | 10.8 | 11.1 | 10.4 | 44 |
| **ToM** |  |  |  |  |
| Brain response | 13.3 | 13.3 | 12.9 | 66 |
| PPI: MTG_STS(L) | 11.8 | 11.7 | 11.4 | 51 |
| PPI: ant. MTS/MTG/ITG(L) | 13.1 | 13.3 | 12.6 | 63 |
| PPI: Amyg/PHG(L) | 10.9 | 10.9 | 10.6 | 43 |
| PPI: PrCu/PCC(L) | 12.4 | 12.3 | 12.0 | 56 |
| PPI: mPFC/ACC/MdFG(R) | 11.8 | 11.8 | 11.7 | 49 |
| PPI: ACC(R) | 11.4 | 12.1 | 11.0 | 52 |
| PPI: MTG/STS(R) | 12.1 | 12.3 | 11.6 | 53 |

Abbreviations: gPPI – generalized psycho-physiological interaction, FWHM – Full Width at Half Maximum; CS – cluster size.

**Supplementary Table 6: Reduced brain response on reward announcing cues in the ASD patient** during a monetary incentive delay (MID) task compared with a matched healthy control group. Statistical threshold is p < .05 cluster size corrected for multiple comparisons (minimum cluster size = 65 voxels).

| **Brain structure: sub region** | **H** | **Cluster size (vox)** | **T(13)** | **p** | **MNI coord. (mm)** | | |
| --- | --- | --- | --- | --- | --- | --- | --- |
|  |  |  |  |  | **x** | **y** | **z** |
|  |  |  |  |  |  |  |  |
|  |  |  |  |  |  |  |  |
| Calcarine Gyrus: V1 | L | 643 | -4.39 | <.001 | -15 | -70 | 6 |
| Lingual Gyrus: V2 | R |  | -5.97 | <.001 | 24 | -49 | 0 |
| Cuneus: V3 | R |  | -4.01 | <.001 | 0 | -85 | 27 |
| Fusiform Gyrus | R |  | -4.18 | <.001 | 36 | -43 | -9 |
| Precuneus | L |  | -4.54 | <.001 | -21 | -49 | 3 |
|  |  |  |  |  |  |  |  |
| Insula: long gyrus | R | 229 | -8.82 | <.001 | 42 | -1 | -9 |
| Insula: anterior part/short gyri | R |  | -4.84 | <.001 | 45 | 14 | -6 |
| Insula/Inferior Frontal Gyrus: orbital part | R |  | -4.30 | <.001 | 45 | 20 | -9 |
| Superior Temporal Gyrus | R |  | -5.73 | <.001 | 48 | 2 | -9 |
|  |  |  |  |  |  |  |  |
| Cerebellum: Lobule VIIa crusII Hemisphere | L | 229 | -4.44 | <.001 | -3 | -82 | -39 |
| Cerebellum: Lobule VIIa crusII Vermis | R |  | -3.81 | .001 | 3 | -82 | -36 |
| Cerebellum: Lobule VIIb Hemisphere | R |  | -3.87 | <.001 | 12 | -73 | -48 |
|  |  |  |  |  |  |  |  |
| Supra Marginal Gyrus | R | 203 | -5.38 | <.001 | 63 | -31 | 48 |
| Inferior Parietal Lobule | R |  | -4.26 | <.001 | 51 | -34 | 48 |
|  |  |  |  |  |  |  |  |
| Superior Temporal Gyrus: TE3 | L | 174 | -5.18 | <.001 | -60 | -7 | -3 |
| Rolandic Operculum: BA44 | L |  | -3.31 | .003 | -48 | 8 | 0 |
|  |  |  |  |  |  |  |  |
| Insula: anterior part/short gyri | L | 133 | -6.37 | <.001 | -30 | 26 | 6 |
| Inferior Frontal Gyrus: orbital part | L |  | -3.79 | .001 | -33 | 20 | -18 |
|  |  |  |  |  |  |  |  |
| Middle Temporal Gyrus | R | 119 | -5.07 | <.001 | 66 | -46 | 0 |
|  |  |  |  |  |  |  |  |
| Posterior Cingulate Cortex | R | 114 | -4.08 | <.001 | 6 | -34 | 48 |
| Paracentral Lobule | R |  | -3.88 | <.001 | 15 | -37 | 54 |
| Precuneus/Superior Parietal Lobule | L |  | -3.30 | .003 | -6 | -49 | 57 |
|  |  |  |  |  |  |  |  |
| Precuneus | R | 114 | -6.02 | <.001 | 6 | -49 | 12 |
|  |  |  |  |  |  |  |  |
| Middle Temporal Gyrus | R | 81 | -3.61 | .002 | 60 | 2 | -21 |
| Inferior Temporal Gyrus | R |  | -3.58 | .002 | 60 | -7 | -27 |
| Medial Temporal Pole | R |  | -3.57 | .002 | 57 | 8 | -21 |
|  |  |  |  |  |  |  |  |
| Middle Occipital Gyrus/Lateral Occipital Cortex | L | 72 | -4.54 | <.001 | -51 | -76 | 18 |
| Middle Temporal Gyrus | L |  | -4.41 | <.001 | -57 | -67 | 12 |
|  |  |  |  |  |  |  |  |

***Abbreviations:*** *H – hemisphere, MNI – Montreal Neurological Institute reference space L – left, R – right, BA – Brodmann area, V – visual cortex, TE – auditory cortex.*

**
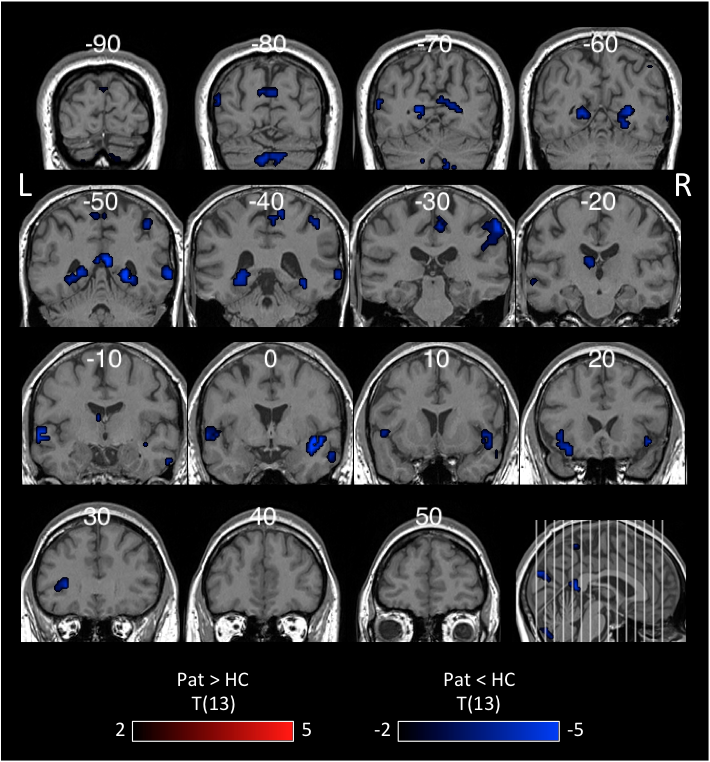
**

**Supplementary Figure 1: Differences in brain response on reward announcing cues in the ASD patient** during a monetary incentive delay (MID) task compared with a matched healthy control group overlaid on coronar slices of ASD patient’s brain in MNI-space. Corresponding y-coordinates are displayed on top of each slice. Regions with elevated brain response are shown in black-red. Regions with reduced brain response are shown in black-blue. All results are p <.05 cluster size corrected (65 voxels). Abbreviations: L – left, R – right, Pat – patient, HC – healthy control group.

**Supplementary Table 7: Elevated brain response on working memory demand in the ASD patient** during a spatio-numerical N-back task compared with a matched healthy control group. Statistical threshold is p < .05 cluster size corrected for multiple comparisons (minimum cluster size = 72 voxels).

| **Brain structure: sub region** | **H** | **Cluster size (vox)** | **T(13)** | **p** | **MNI  coord. (mm)** | | |
| --- | --- | --- | --- | --- | --- | --- | --- |
|  |  |  |  |  | **x** | **y** | **z** |
|  |  |  |  |  |  |  |  |
|  |  |  |  |  |  |  |  |
| Posterior-Medial Frontal | L | 1286 | 5.66 | <.001 | -9 | -10 | 57 |
| Paracentral Lobule: BA4a | L |  | 4.80 | <.001 | -3 | -28 | 57 |
| Middle Frontal Gyrus: posterior part | L |  | 4.74 | <.001 | -36 | -1 | 57 |
| Superior Frontal Gyrus | L |  | 4.64 | <.001 | -18 | -10 | 75 |
|  |  |  |  |  |  |  |  |
| Ventral Striatum | L | 539 | 5.50 | <.001 | -6 | 2 | -9 |
| Ventral Striatum | R |  | 4.11 | <.001 | 9 | 11 | -9 |
| Thalamus: Prefrontal | L |  | 4.87 | <.001 | -3 | -16 | -3 |
| Thalamus: Prefrontal | R |  | 3.27 | .003 | 12 | -19 | 6 |
| Thalamus: Somatosensory | R |  | 3.30 | .003 | 12 | -22 | 0 |
| Thalamus: Premotor | R |  | 3.27 | .003 | 21 | -10 | -9 |
| Hypothalamus: anterior part | R |  | 3.72 | .001 | 3 | -1 | -9 |
|  |  |  |  |  |  |  |  |
| Lingual Gyrus: V1 | R | 281 | 3.84 | .001 | 12 | -82 |  |
| Calcarine Gyrus: V1 | R |  | 3.53 | .002 | 12 | -91 | 3 |
| Superior Occipital Gyrus: V3 | R |  | 4.80 | <.001 | 21 | -88 | 18 |
| Middle Occipital Gyrus | R |  | 4.15 | <.001 | 30 | -79 | 15 |
|  |  |  |  |  |  |  |  |
| Precentral Sulcus/Premotor cortex | R | 266 | 4.75 | <.001 | 21 | -1 | 48 |
| Middle Frontal Gyrus: posterior part | R |  | 4.25 | <.001 | 30 | -7 | 54 |
| Postcentral Gyrus: BA3b | R |  | 3.26 | .003 | 42 | -19 | 48 |
| Superior Frontal Gyrus | R |  | 4.24 | <.001 | 30 | -7 | 66 |
|  |  |  |  |  |  |  |  |
| Precentral gyrus: inferior part, BA44 | R | 121 | 6.17 | <.001 | 54 | 5 | 18 |
|  |  |  |  |  |  |  |  |
| Inferior Frontal Gyrus: triangular part | L | 74 | 3.47 | .002 | -33 | 29 | 3 |
| Putamen | L |  | 3.32 | .003 | -24 | 14 | 0 |
| Insula: anterior part, short gyri | L |  | 3.24 | .003 | -30 | 17 | 3 |
|  |  |  |  |  |  |  |  |

***Abbreviations:*** *H – hemisphere, MNI – Montreal Neurological Institute reference space L – left, R – right, BA – Brodmann area, V – visual cortex.*

**Supplementary Table 8: Reduced brain response on working memory demand in the ASD patient** during a spatio-numerical N-back task compared with a matched healthy control group. Statistical threshold is p < .05 cluster size corrected for multiple comparisons (minimum cluster size = 72 voxels).

| **Brain structure: sub region** | **H** | **Cluster size (vox)** | **T (13)** | **p** | **MNI  coord. (mm)** | | |
| --- | --- | --- | --- | --- | --- | --- | --- |
|  |  |  |  |  | **x** | **y** | **z** |
|  |  |  |  |  |  |  |  |
|  |  |  |  |  |  |  |  |
| Cerebellum: Lobule VIIa crusII Hemisphere | L | 339 | -4.80 | <.001 | -12 | -82 | -36 |
| Cerebellum: Lobule VIIa crusI Hemisphere | L |  | -3.61 | .002 | -33 | -85 | -27 |
|  |  |  |  |  |  |  |  |
| Cerebellum: Lobule VIIa crusI Hemisphere | R | 190 | -5.42 | <.001 | 45 | -76 | -33 |
| Cerebellum: Lobule VIIa crusII Hemisphere | R |  | -4.30 | <.001 | 12 | -82 | -36 |
|  |  |  |  |  |  |  |  |
| Inferior Temporal Gyrus | R | 122 | -4.12 | <.001 | 57 | -28 | -15 |
| Middle Temporal Gyrus | R |  | -3.53 | .002 | 69 | -31 | -12 |
|  |  |  |  |  |  |  |  |

***Abbreviations:*** *H – hemisphere, MNI – Montreal Neurological Institute reference space L – left, R – right.*

**
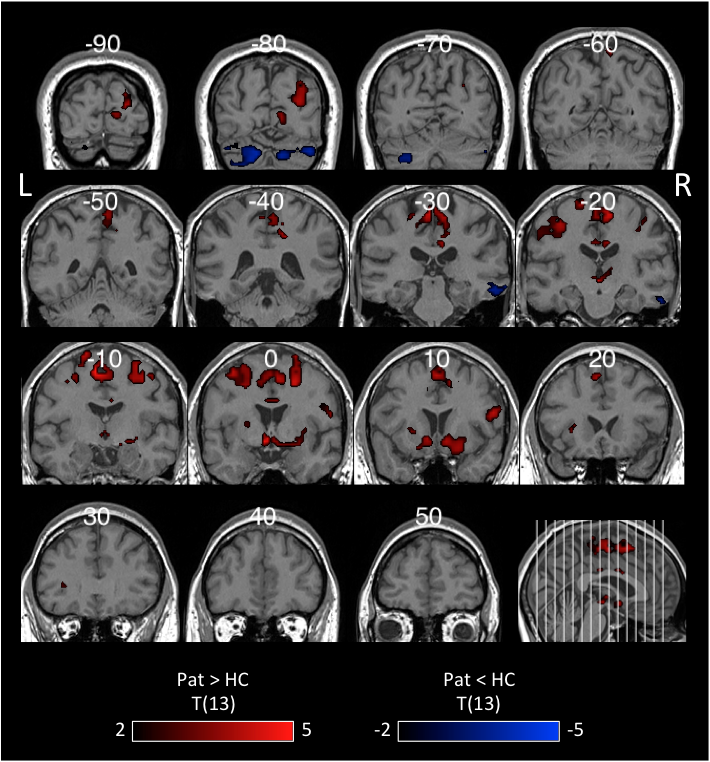
**

**Supplementary Figure 2: Differences in brain response on memory demand in the ASD patient** during a spatially-numerically N-back task compared with a matched healthy control group overlaid on coronar slices of ASD patients’ brain in MNI-space. Corresponding y-coordinates are displayed on top of each slice. Regions with elevated brain response are shown in black-red. Regions with reduced brain response are shown in black-blue. All results are p <.05 cluster size corrected (72 voxels). Abbreviations: L – left, R – right, Pat – patient, HC – healthy control group.

**Supplementary Table 9: Elevated brain response during a face-matching task in the ASD patient** compared with a matched healthy control group. Statistical threshold is p < .05 cluster size corrected for multiple comparisons (minimum cluster size = 69 voxels).

| **Brain structure: sub region** | **H** | **Cluster size (vox)** | **T (13)** | **p** | **MNI  coord. (mm)** | | |
| --- | --- | --- | --- | --- | --- | --- | --- |
|  |  |  |  |  | **x** | **y** | **z** |
|  |  |  |  |  |  |  |  |
|  |  |  |  |  |  |  |  |
| Calcarine Gyrus: V1 | L | 246 | 5.01 | <.001 | -18 | -73 | 9 |
| Lingual Gyrus: V1 | L |  | 4.80 | <.001 | -18 | -64 | 3 |
| Lingual Gyrus: V3 | L |  | 3.65 | .001 | -15 | -61 | -9 |
|  |  |  |  |  |  |  |  |
| Cerebellum: Lobule VIIa crusI Hemisphere | L | 121 | 8.89 | <.001 | -30 | -85 | -24 |
|  |  |  |  |  |  |  |  |
| Cerebellum: Lobule VI Hemisphere | L | 79 | 4.67 | <.001 | -30 | -61 | -24 |
| Cerebellum: Lobule VIIa crusI Hemisphere | L |  | 3.61 | .002 | -39 | -58 | -30 |
|  |  |  |  |  |  |  |  |

***Abbreviations:*** *H – hemisphere, MNI – Montreal Neurological Institute reference space L – left, R – right, V – visual cortex.*

**
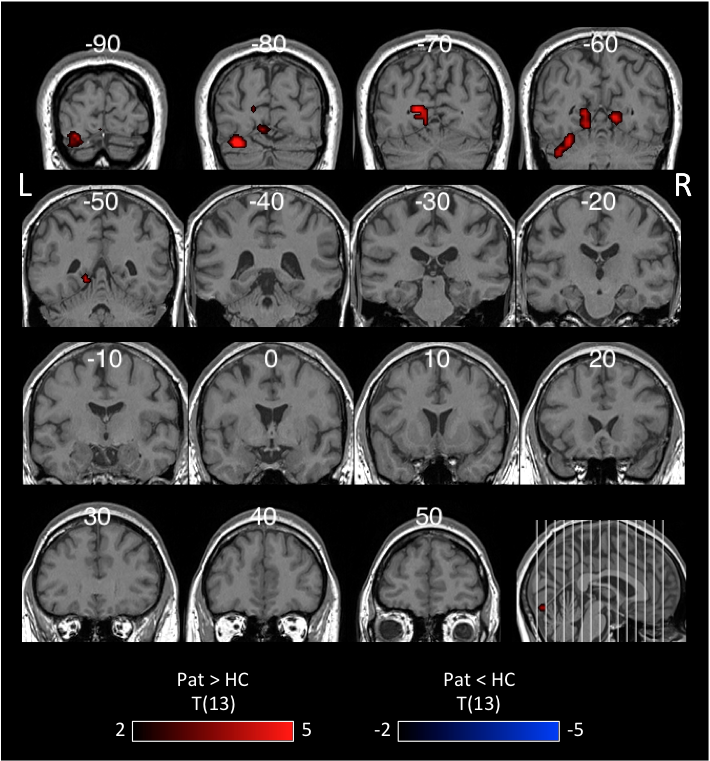
**

**Supplementary Figure 3: Differences in brain response during face perception in the ASD patient** during a face-matching task compared with a matched healthy control group overlaid on coronar slices of ASD patients’ brain in MNI-space. Corresponding y-coordinates are displayed on top of each slice. Regions with elevated brain response are shown in black-red. Regions with reduced brain response are shown in black-blue. All results are p <.05 cluster size corrected (69 voxels). Abbreviations: L – left, R – right, Pat – patient, HC – healthy control group.
